# Mitochondrial Membrane Dynamics—Functional Positioning of OPA1

**DOI:** 10.3390/antiox7120186

**Published:** 2018-12-08

**Authors:** Hakjoo Lee, Yisang Yoon

**Affiliations:** Department of Physiology, Medical College of Georgia, Augusta University, Augusta, GA 30912, USA; haklee@augusta.edu

**Keywords:** mitochondria, mitochondrial dynamics, fission, fusion, Oxidative phosphorylation (OXPHOS), OPA1, cristae, Overlapping activity with m-AAA protease 1 (OMA1), cardiolipin

## Abstract

The maintenance of mitochondrial energetics requires the proper regulation of mitochondrial morphology, and vice versa. Mitochondrial dynamins control mitochondrial morphology by mediating fission and fusion. One of them, optic atrophy 1 (OPA1), is the mitochondrial inner membrane remodeling protein. OPA1 has a dual role in maintaining mitochondrial morphology and energetics through mediating inner membrane fusion and maintaining the cristae structure. OPA1 is expressed in multiple variant forms through alternative splicing and post-translational proteolytic cleavage, but the functional differences between these variants have not been completely understood. Recent studies generated new information regarding the role of OPA1 cleavage. In this review, we will first provide a brief overview of mitochondrial membrane dynamics by describing fission and fusion that are mediated by mitochondrial dynamins. The second part describes OPA1-mediated fusion and energetic maintenance, the role of OPA1 cleavage, and a new development in OPA1 function, in which we will provide new insight for what OPA1 does and what proteolytic cleavage of OPA1 is for.

## 1. Introduction

Mitochondria are the essential membrane organelles in eukaryotic cells, producing the energy necessary for a multitude of cellular processes and cell survival. Frequent changes in the shape and location of mitochondria inside cells have been observed and are termed ‘mitochondrial dynamics’. Mitochondrial shape change occurs mainly through fission and fusion. Mitochondria are unique in that they are double-membraned organelles and include elaborate internal membranes; therefore, mitochondrial membrane dynamics is a complicated process that requires the coordinated regulation of protein and lipid molecules. Oxidative phosphorylation (OXPHOS) is the mitochondrial adenosine triphosphate (ATP)-generating process, which affects, and is affected by, mitochondrial fission and fusion, which demonstrates the intimate relationship between mitochondrial morphology and energetic activity. The protein, optic atrophy 1 (OPA1), is a membrane remodeling protein that regulates both mitochondrial morphology and energetics. In the following sections, we will describe the roles of proteins and lipids in mitochondrial membrane dynamics and the recent development regarding the mechanisms by which OPA1 regulates mitochondrial shape and OXPHOS.

## 2. Structural Organization of Mitochondria

**Figure 1:**
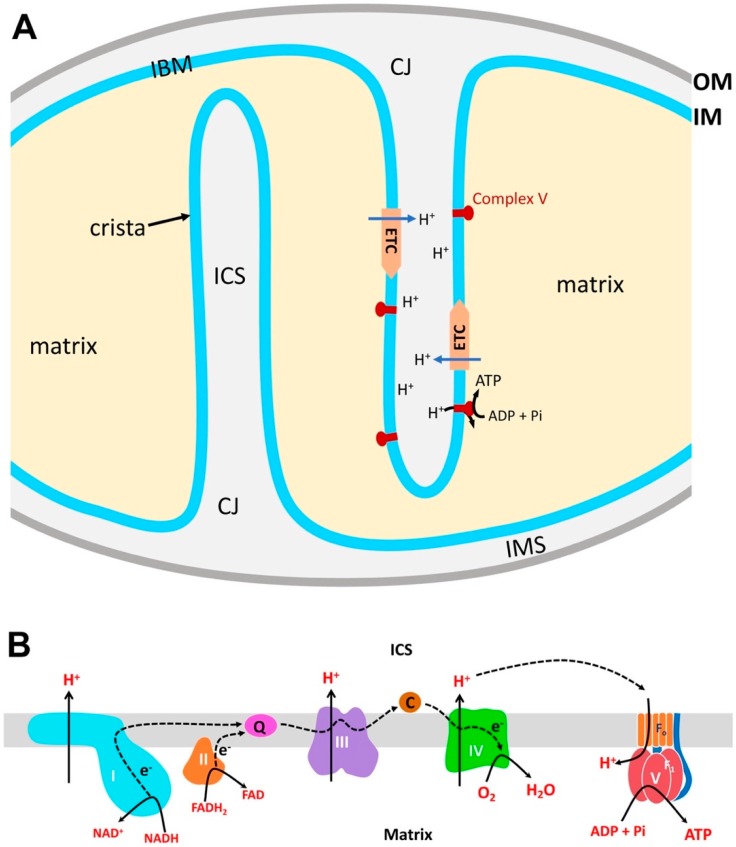
Mitochondrial compartments and electron transport chain (ETC). (**A**) OM, outer membrane; IM, inner membrane; IMS, intermembrane space; IBM, inner boundary membrane; ICS, intra-cristal space; CJ, cristae junction. In matrix, the reducing equivalents are generated from substrate oxidation (TCA cycle, fatty acid oxidation, and other metabolisms) and donate electrons to ETC for proton translocation to ICS. Re-entering of protons to the matrix through complex V drives ATP synthesis. (**B**) Electron flow in the ETC. Coenzyme Q (Q) and cytochrome *c* (C) are mobile electron carriers.

Mitochondrial membranes are critical for ATP production, because OXPHOS requires membrane-associated enzymes and regulated exchanges of ions and metabolites through membrane channels, transporters, and carriers. Two lipid bilayers of mitochondrial membranes, the inner membrane (IM) and the outer membrane (OM), enclose the inner soluble parts of the mitochondria termed matrix and intermembrane space (IMS), respectively (Figure 1A). The major part of the IM is the cristae, the invaginated membrane harboring membrane proteins, such as the electron transport chain (ETC) complexes. Cristae can be lamellar, likely formed by infoldings of the IM sheet, or tubular [1,2,3]. The intra-cristal space (ICS) is the inside volume of lamellar or tubular cristae, continuous with the IMS; however, the functional continuity is thought to be limited by the narrow cristae junction (CJ) structure. The rest of the IM that runs parallel with the OM is called the inner boundary membrane (IBM).

OXPHOS is the coupled event of nutrient oxidation and adenosine diphosphate (ADP) phosphorylation [4,5]. Metabolic intermediates are transported to the mitochondrial matrix where they are oxidized to generate the reducing equivalents NADH (nicotinamide adenine dinucleotide, reduced) and FADH_2_ (flavin adenine dinucleotide, reduced). Electrons from the reducing equivalents that are generated in the matrix pass through the ETC complexes (complexes I–IV) embedded in the cristae membrane (Figure 1A). Electrons from NADH are passed on to complex I and flow to complex III and IV. Complex II generates FADH_2_ and passes electrons to complex III and complex IV (Figure 1B). At complex IV, electrons reduce oxygen to generate water. Through electron transport along the ETC, complexes I, III, and IV pump out protons to the ICS, creating the proton motive force across the cristae membrane (Figure 1B). Although the ICS is spatially continuous with the IMS, protons in the ICS are unlikely to diffuse into the IMS due to the CJ structure. ATP synthase (complex V; F_o_F_1_-ATPase) is embedded in the cristae membrane by the F_o_ part, and its active site (F_1_ complex) is in the matrix. Proton translocation from ICS to the matrix through the F_o_ rotates the c-ring of F_o_, by which ADP and phosphate are combined in the F_1_ active site for ATP synthesis (Figure 1A,B). Within the cristae membrane, ETC complexes and ATP synthase can further assemble to form supercomplexes [6,7]. While the composition of supercomplexes varies, the most notable supercomplex is the “respirasome”, which is composed of complexes I, III, and IV [8,9]. The supercomplex assembly of the ETC complexes is thought to promote efficient electron transport [10,11]. Complex V can also form dimers and oligomers that are involved in inducing IM curvature for cristae formation for increased ATP production [6,12,13]. The structure of cristae may also influence OXPHOS activity [1,2,3]. As mentioned, typical cristae are lamellar or tubular, and the mixture of both is also found in one mitochondrion [1,3]. Furthermore, in most cristae in neuronal mitochondria, a single crista contains both lamellar and tubular segments [14]. Although the functional difference between the two types of cristae is unclear, two forms may be inter-convertible depending on energetic demand. In cardiac muscle cells, tubular cristae are abundant in interfibrillar mitochondria that have higher OXPHOS activity, whereas mostly lamellar cristae are found in energetically less active subsarcolemmal mitochondria [1]. These observations suggest that tubular cristae may be suited for enhanced ATP synthesis, possibly by the easier formation of proton gradient due to the smaller ICS volume [1]. The elaborate processes of electron transport and ATP synthesis require directional movements of metabolites, electrons, and ions through the specific mitochondrial compartments; therefore, the structural organization of mitochondria is tailored for the efficient production of ATP.

## 3. Proteins and Lipids for Mitochondrial Membrane Remodeling

### 3.1. Mitochondrial Dynamins

Dynamin proteins are phylogenetically conserved proteins that remodel biological membranes by GTPase activity. Mitochondrial fission and fusion are mediated by a group of dynamin proteins. They are dynamin-like/related protein1 (DLP1/Drp1), mitofusin1 (Mfn1), mitofusin2 (Mfn2), and optic atrophy1 (OPA1). Because many reviews have been written about the detailed mechanisms and roles of these proteins in pathophysiology [15,16,17,18], here we will provide a brief synopsis about these dynamins.

**Figure 2:**
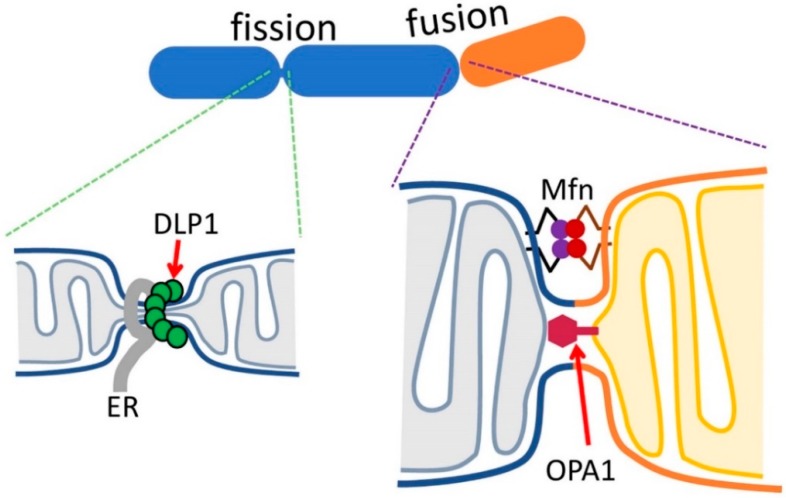
Mitochondrial fission and fusion by dynamin-related proteins. The ER (Endoplasmic reticulum) constricts mitochondria through a contractile force exerted from actin, myosin, and their associated proteins. DLP1 assembles at the constriction with the aid of its receptor and mediates fission through GTP hydrolysis. Final scission may require Dyn2. Mfn may interact in a head-to-head manner to tether and fuse opposing OMs. IM-anchored OPA1 may bind to the opposing IM for fusion.

DLP1 is the mitochondrial fission protein that is cytosolic and is recruited to the mitochondrial outer surface where it assembles into ring-like structures for fission [19] (Figure 2). While in vitro studies using recombinant DLP1 showed that it binds directly to the membrane for membrane remodeling [20,21,22,23,24], in cells, there are receptor/adaptor proteins at the mitochondrial surface for regulated mitochondrial recruitment of DLP1. Mitochondrial fission factor (Mff) and mitochondrial dynamics proteins of 49 and 51 kDa (MiD49 and MiD51) have been established as DLP1 receptors with Mff as the primary receptor [21,25,26,27,28,29,30,31,32]. Fis1, originally considered to be a DLP1 receptor [33,34,35], was found to have a less of a role as a DLP1 receptor [21,29,30,31,32], though it may participate in the stress-induced fission of mitochondria destined to mitophagy [36]. Endoplasmic reticulum (ER) tubules have been shown to participate in the initial constriction of the mitochondrial tubules to determine sites where DLP1 is recruited for fission [37] (Figure 2). Actin, actin-modulating proteins, and myosin have been identified to be involved in generating contractile force for ER-mediated mitochondrial constriction [16,38,39,40]. Additionally, silencing conventional dynamin (Dyn2) was shown to elongate mitochondria similarly to a DLP1 deficiency, suggesting its involvement in mitochondrial fission [41]. It was found that Dyn2 is recruited to the mitochondrial constriction site that was created by DLP1, where it mediates the final separation of the mitochondrial membrane for fission [41].

Two isoforms of mitofusin, Mfn1 and Mfn2, are the OM fusion dynamins. Mfn1 and Mfn2 can form homomeric and heteromeric complexes [42]. Mfns have two transmembrane (TM) domains at the C-terminus that are presumed to span the OM twice, positioning both N-terminal domains (GTPase domain and heptad repeat 1 [HR1] domains) and the C-terminal tail (heptad repeat 2 [HR2]) at the cytosolic face of mitochondria [43]. Mfn molecules are required to be present on both partner membranes for OM fusion [43] (Figure 2). The crystal structure of Mfn1 C-terminal HR2 revealed dimeric anti-parallel coiled coil, suggesting that the tethering of fusing mitochondria is mediated by an HR2-HR2 interaction at the cytosolic face of partner mitochondria [43]. However, other studies indicated that the HR2-HR2 interaction may not occur [44], and suggested that tethering may be mediated by the interaction between GTPase domains [44,45]. Furthermore, a recent report suggests that Mfns are single TM proteins with the C-terminal HR2 in the IMS [46]. This study indicated that Mfn oligomerizes through the redox-mediated disulfide modifications at the IMS to drive fusion. Cryo-electron tomography of yeast mitochondria revealed stunning images of tethering and docking of OMs that precede OM fusion [47]. Protein density was found in the gap between the OMs of tethered mitochondria, and the periphery of the OM contact of docked mitochondria (docking ring), suggesting the involvement of Fzo1 protein (yeast Mfn homologue) in the OM tethering and docking processes. A fusion pore was found at the periphery of the docked OMs, where the protein density in the docking ring was sparse [47]. These observations suggest that OM fusion is accompanied by Fzo1/Mfn dissociation, which may facilitate the fusion pore expansion for full fusion, possibly by removing steric hindrance of rigid protein structures in the fusion site.

OPA1 was first identified as a human disease gene that causes autosomal dominant optic atrophy when mutated [48,49]. It is required for IM fusion following Mfn-mediated OM fusion (Figure 2). OPA1 expression goes through alternative splicing, as well as proteolytic processing, generating multiple variant forms that include IM-anchored long OPA1 (L-OPA1) and soluble short OPA1 (S-OPA1). OPA1 has been shown to play a role in mitochondrial energetics, presumably by maintaining cristae structure. Several recent studies provide new information about the mechanisms and roles of L- and S-OPA1 in IM fusion and energetic maintenance. OPA1 will be discussed in more detail in a later section.

### 3.2. Cardiolipin, a Crucial Mitochondrial Lipid for Membrane Remodeling and Energetic Maintenance

Phospholipids are the main building blocks of lipid bilayers. Phospholipids generally have two hydrophobic acyl chains and a modified phosphate group that are linked by a glycerol molecule. Plasma membranes and cellular organelles have their own unique lipid composition (review [50]). Within mitochondria, OMs and IMs have different lipid composition and an asymmetric distribution of lipids. Additionally, mitochondrial contact sites between the OM and IM also show different lipid composition from the rest of the mitochondria [50]. Phospholipids are mainly synthesized in the ER and are transported to mitochondria. Mitochondrial phospholipid synthesis is limited to phosphatidic acid, phosphatidylethanolamine, phosphatidylglycerol, and cardiolipin [50]. Cardiolipin (diphosphatidylglycerol, CL) is a unique phospholipid with a dimeric structure containing four acyl chains and two phosphatidyl moieties that are linked to glycerol. CL is exclusively synthesized in the IM and is enriched in the matrix side of the IM [51]; (review [50]). Because of its unique structure with a small anionic head group relative to the four acyl chains, CL is cone-shaped in the lipid bilayer; therefore, CL microdomains form a negative curvature when in an enriched condition in the membrane, and, when further enriched, transition from a lamellar bilayer phase to the non-lamellar, inverted hexagonal (H_II_) configuration [52,53]. The inherent physical property of anionic CL associated with cristae formation has been shown in the lipid-only membrane. Applying a local pH decrease (matrix pH 8 to acidic outside, similar to proton motive force across mitochondrial IM) to a CL-containing minimal membrane vesicle causes spontaneous tubulation that is similar to a tubular crista shape [54]. CL is also enriched in mitochondrial contact sites between the OM and IM probably resulting from its intrinsic property to bend the lipid membrane [55].

The fission and fusion proteins interact with specific lipids of the membrane, including CL (review [56]). CL binding increases cooperative GTPase activity of DLP1 [57,58]. Furthermore, CL on the OM is reorganized by DLP1 and, through its intrinsic property, CL enables DLP1 to divide mitochondria [23]. On the other hand, the DLP1 fission activity is inhibited when DLP1 interacts with fusogenic phosphatidic acid (PA) and an acyl group of a neighboring saturated phospholipid [59]. OM fusion takes place through tethering opposing OMs with Mfn1 or Mfn2. Mitochondrial phospholipase D (MitoPLD) generates fusogenic PA by converting CL on the OM [60]. Thus, mitochondria of MitoPLD-knockout (KO) cells are fragmented while MitoPLD overexpression causes mitochondrial aggregation [60]. A recent report shows that MitoPLD interacts with DLP1 as well as Mfn1, suggesting that MitoPLD supports a compartmentalized lipid environment for promoting fusion and inhibiting fission through the conversion of CL to PA [59]. CL binding also activates GTPase activity of OPA1 [61]. Mgm1, the yeast OPA1 homologue, has a lipid-binding domain residing between the Middle domain and GTPase effector domain, and it binds preferentially to CL, which stimulates Mgm1 GTPase activity [62].

In addition, CL binds and stabilizes various mitochondrial membrane proteins, including each of the OXPHOS complexes and their supercomplexes, adenine nucleotide translocase (ANT), and cytochrome *c* (review [63]). Acyl chains of CL are highly unsaturated, and thus CL is prone to oxidation in oxidative stress conditions, which contributes to cytochrome *c* release. CL-bound cytochrome *c* acts as CL peroxidase and it generates CL hydroperoxides, resulting in cytochrome *c* release from mitochondria [64].

Although CL is enriched in the IM, CL is transferred to the OM by the CL-binding proteins, nucleoside diphosphate kinase-D (NDPK-D, NM23-H4) and mitochondrial creatine kinase (mtCK) [65]. NDPK-D is localized in the IMS where it normally converts GDP to GTP using ATP generated from OXPHOS, and the produced ADP is transported back to the matrix by ANT for ATP synthesis [66,67]. NDPK-D has been shown to provide GTP for the OPA1 GTPase for IM fusion [68]. NDPK-D forms symmetrical homohexamers that can bind CL and other anionic phospholipids, and its phosphotransfer activity requires lipid binding [66]. In addition to its phosphotransfer activity, NDPK-D also assists in intermembrane lipid transfer from IM to OM [67]. When cells are treated with respiration inhibitors, such as rotenone, the phosphotransferase activity is shut off, and NDPK-D instead transfers CL from the IM to the OM [67,69]. The transported CL on the OM then interacts with CL-binding proteins, such as DLP1 for fission, LC3-II for mitophagy [70], and tBid/Bax for apoptosis [71].

## 4. Functional Positioning of OPA1

### 4.1. OPA1 Variants

**Figure 3:**
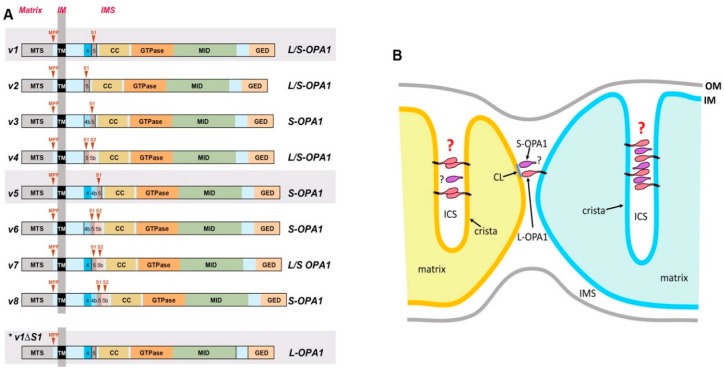
OPA1 variants and operating models for IM fusion and cristae width maintenance. (**A**) Eight different OPA1 variants. Cleavages at S1 and S2 generate S-OPA1. OPA1-v3, v5, v6, v8, which contain the region encoded by exon 4b, are cleaved completely to generate only S-OPA1. The cleavage in the other four variants is incomplete, generating both L- and S-OPA1 (L/S-OPA1). OPA1-v1∆S1 (*) is an experimentally generated mutant in which the S1 site is deleted in the OPA1-v1 variant, and thus forms only L-OPA1. OPA1-v1, v5, and v1∆S1 (shaded) were used as representatives for L/S-, S-, and L-OPA1, respectively, in our study [96]. MPP, mitochondrial processing peptidase. (**B**) IM anchored L-OPA1 binds to CL of opposing IM for tethering and subsequent fusion, and S-OPA1 may facilitate this process. Cristae tightness may be maintained by trans-interaction of L-OPA1 (left crista) or by L-OPA1 oligomerization that is facilitated by S-OPA1 functioning as molecular staples (right crista). However, these models have recently been challenged by a new finding that S-OPA1 can maintain cristae tightness.

The protein OPA1 is encoded by a single gene that contains total 30 exons. Alternative splicing involving exons 4, 4b, and 5b generates eight different mRNA species in humans [72,73,74,75] (Figure 3A). The expression pattern of these mRNA species varies in different tissues [76]. All eight precursor proteins, produced from translation of the eight mRNAs in the cytosol, contain the N-terminal mitochondrial transit sequence (MTS) that enables mitochondrial import. The TM domain downstream of the MTS acts as a stop-transfer signal, anchoring the OPA1 protein at the IM, and leaving most of the protein in the IMS [77] (Figure 3A).

After mitochondrial import, the membrane-anchored OPA1 (“L-OPA1” for long form) can be cleaved by proteases downstream from the TM region, generating TM-free OPA1 (“S-OPA1” for short form) that is peripherally bound to the IM in the IMS. Two cleavage sites, S1 and S2, each cleaved by the IM-associated metalloproteases OMA1 and Yme1L, respectively, have been identified [73,74,78,79,80] (Figure 3A). All eight OPA1 variants have the region encoded by exon 5 where the S1 site is, whereas four of the variants contain the S2 site in the exon 5b-encoded region in addition to S1 (Figure 3A). It appears that the presence of the region encoded by exon 4b is the prerequisite for complete cleavage at S1, which generates S-OPA1 exclusively (v3, v5, v6, and v8). Without the exon 4b-encoded sequence, the cleavage at S1 is incomplete, resulting in both uncleaved L-OPA1 and cleaved S-OPA1. Exon 4b is predicted to encode a hydrophobic patch that possibly penetrates the IM, which may be required for synergistic activation of IM-embedded OMA1 for efficient cleavage at S1. S-OPA1 generated by S1 or S2 cleavage contains functional domains of dynamin proteins (GTPase domain, Middle domain, and GTPase effector domain GED) (Figure 3A). Two additional OPA1 species smaller than S-OPA1 were observed in postprandial livers [81]. These atypical OPA1 species are generated by cleavages that inactivate GTPase activity, indicating the cleavage-mediated loss of OPA1 function [81].

### 4.2. Multiple Roles of OPA1

OPA1 is well-known as the IM fusion protein. Indeed, OPA1-KO cells frequently reveal two or more matrix compartments that are enclosed in a single OM, indicating that OPA1 is required for IM fusion following Mfn-mediated OM fusion [82]. Interestingly, S-OPA1 was proposed to be involved in IM fission [83], but further verification is necessary. In addition to the IM fusion function, OPA1-silencing or KO increases apoptotic sensitivity and decreases the mitochondrial DNA (mtDNA) level/stability and energetic activity [84,85,86,87,88,89].

During apoptosis, OPA1 has been suggested to regulate cytochrome *c* release from the ICS. The report from Scorrano’s group indicated that OPA1 oligomerizes to keep the CJ tight and that, during tBid-induced apoptosis, OPA1 complexes become dissociated to widen the CJ, allowing for the release of cytochrome *c* from the ICS [90]. However, other studies showed either no change in CJ diameter or even narrower CJ during apoptosis [91,92]. Regardless of whether the CJ is altered or not, it was shown that OPA1 disassembly is necessary for cytochrome *c* release during apoptosis [90,92]. Hence, it is likely that the OPA1 assembly state, not CJ size, is a key factor for cytochrome *c* release. Possibly, assembled OPA1 at the CJ or the neck area of cristae acts as a barrier and OPA1 dissociation allows for cytochrome *c* release [93,94].

Immunoelectron microscopy showed that OPA1 is not enriched in the CJ; rather, it is mostly associated with the cristae membrane and IBM [77,85,95]. Typical immunoEM for OPA1 shows only a few gold particles in mitochondria. By overlaying the locations of gold particles from numerous OPA1-immunoEM images on mitochondria, OPA1 was found more enriched along the central neck area of cristae with gradual decreases toward CJ and cristae tip [95]. The most striking ultrastructural defect in OPA1 deficiency is the disruption of cristae structure, showing greatly decreased cristae number and the loss of cristae tightness [84,85,96]. The cristae “tightness” depicts the narrowness of the width between two cristae membranes forming the ICS in a two-dimensional plane. OPA1 deficiency causes defects in respiration and respiratory supercomplex assembly, likely due to the loss of cristae membrane in which respiratory complexes and supercomplexes assemble [87,96,97,98]. The observations that OPA1 deficiency decreases cristae density suggest that OPA1 is involved in the biogenesis or maintenance of cristae membranes. While the mechanism by which OPA1 regulates cristae density is not understood, mild overexpression of OPA1 has been shown to enhance supercomplex assembly and respiration efficiency by increasing cristae tightness [97,99], indicating that cristae tightness is a critical factor for maintaining energetic efficiency. It is likely that the functions of OPA1 in energetic maintenance and apoptotic resistance are attributed to the structural role of OPA1 for cristae biogenesis and maintaining cristae tightness [96,97,99].

Another interesting role of OPA1 was reported in adipocytes where it serves as an A-kinase anchoring protein (AKAP) on the surface of lipid droplets (LDs) [100]. In this role, OPA1 was shown to localize to the LD surface and bind to protein kinase A (PKA), which phosphorylates the LD protein perilipin to promote lipolysis during adrenergic stimulation. However, several questions were raised for the experimental tools that were used in the study and the mechanism of OPA1 localization on LD [101]. While colocalization and co-immunoprecipitation of endogenous OPA1 with LDs, perilipin, and PKA were shown [100], some of the colocalization studies used GFP-fused OPA1 at the N-terminus, which might have disrupted the N-terminal mitochondrial targeting information of OPA1. Additionally, although OPA1 silencing decreased lipolysis in 3T3-L1 adipocytes, rescue experiments used overexpression of the same incorrect OPA1 fusion constructs [100]. More recently, the same group used a proximity ligation assay to show the close proximity of OPA1 and LD surface proteins perilipin 1 and CGI-58, supporting earlier findings [102]. Previous studies showed that perilipin 5 recruits mitochondria to LDs [103], and more recently that perilipin 1 of LDs binds to Mfn2 of mitochondria in brown adipose tissue, allowing for the docking of the two organelles [104]. This subpopulation of mitochondria, now dubbed peridroplet mitochondria (PDM), are tightly associated with the LDs for metabolic communication [105]. Whether OPA1 on LDs is a definitive finding or a spurious observation of PDM OPA1 will need further verification.

### 4.3. Current Models for OPA1-Mediated Fusion and Maintenance of Cristae Tightness

Mitochondrial fusion assays by forming hybrids between OPA1-KO and wild-type cells, each having a different fluorophore in the mitochondrial matrix, showed the mixing of two fluorophores in the mitochondria of hybrid cells, demonstrating that the presence of OPA1 in only one of the fusing mitochondria is sufficient for IM fusion [82]. In addition, it was shown that the presence of both L- and S-OPA1 is necessary for mitochondrial fusion, while L- or S-OPA1 alone is insufficient for fusion [74]. However, other studies showed that L-OPA1 alone is fusion competent while S-OPA1 is not [73,83,96,106]. Regarding OPA1’s role for maintaining cristae tightness, OPA1 cleavage or disassembly of the OPA1 oligomer in tBid-induced apoptosis causes the loss of cristae tightness for cytochrome *c* release [90,107]. Furthermore, OMA1/Yme1L-double KO (DKO) cells containing only L-OPA1 maintain cristae tightness [83], indicating the sufficient role of L-OPA1 for cristae maintenance. Hence, a currently suggested model states that L-OPA1 anchored at the cristae membranes interacts with each other to tether and keep the cristae membrane close. Although S-OPA1 is not strictly required, it was suggested to function as molecular staples, binding to L-OPA1 to increase OPA1 oligomerization [90,107] (Figure 3B, right crista).

Recent in vitro studies using a liposome fusion assay with recombinant OPA1 reconstituted in the liposome showed that membrane-anchored L-OPA1 binds to CL of the opposing membrane to mediate tethering and subsequent fusion [108] (Figure 2B). It was shown that efficient fusion can occur between OPA1-containing liposomes and CL-rich liposomes that do not contain OPA1, supporting the finding from the cell study that the presence of OPA1 in one of the fusing partners is sufficient for IM fusion. CL was critical for fusion, as increasing CL content enhanced fusion, while no fusion was detected with no or low CL content. Furthermore, adding S-OPA1 to the L-OPA1-containing fusion mixture increased fusion efficiency [108], as is consistent with efficient fusion in the presence of both L- and S-OPA1, as observed in cells [74]. Using a liposome tethering assay, it was shown that L-OPA1-containing liposomes that contained no or low concentration of CL were tethered together by forming trans-complexes of L-OPA1. With insufficient CL, these tethered liposomes did not undergo fusion [108]. This study also found that GTP hydrolysis is required for membrane fusion, but not tethering. This series of observations led to the model of IM fusion and maintenance of cristae tightness mediated by OPA1. IM fusion occurs at an OM-IM contact site that contains high CL content: IM anchored L-OPA1 binds to CL of the opposite IM for tethering and subsequent fusion by GTP hydrolysis [108] (Figure 3B). On the other hand, the cristae membrane may have less CL than contact sites [55]; therefore, trans-interacting L-OPA1 in the ICS would maintain cristae tightness [108] (Figure 3B, left crista). While this model can largely explain the observations that were made in cell studies, this in vitro experimental system presented shortcomings, as robust fusion was observed when S-OPA1 was mixed with CL-rich liposomes [109], contradicting the observations made in cells. The role of S-OPA1 in keeping cristae tight, as molecular staples needs to be further addressed in this model. Furthermore, a previous report indicated that OPA1 cleavage is a driving event for IM fusion [110] (see below). Testing this model will need a different in vitro experimental system.

### 4.4. OPA1 Cleavage at S1, a Bad Omen of Mitochondrial Demise?

Stress conditions likely associated with mitochondrial dysfunction activate OMA1 to cleave OPA1 at S1, which causes mitochondrial fragmentation by fusion inactivation [79,80,111]. The S1 cleavage of OPA1 can segregate dysfunctional mitochondria by preventing fusion, and would facilitate autophagic removal of them (mitophagy) [112,113]. In addition to fusion inactivation, the loss of L-OPA1 and the accumulation of S-OPA1 by OMA1-mediated cleavage have been shown to disrupt cristae structure and render apoptotic sensitivity, suggesting that OPA1 cleavage at S1 is detrimental to cell survival [79,83,114]. 

On the other hand, Yme1L-mediated OPA1 cleavage has been shown to drive mitochondrial fusion upon OXPHOS stimulation [110]. These observations suggest that OPA1 cleavage at S1 and S2 may have different functional outcomes, in which OMA1-mediated cleavage is associated with mitochondrial and cell death, whereas Yme1L-mediated cleavage is associated with mitochondrial maintenance. However, mitochondrial fusion mediated by OPA1 cleavage was not limited to Yme1L-specific cleavage, as nonspecifically engineered cleavage of OPA1 was sufficient to mediate IM fusion [110]. The “OPA1 cleavage-mediated fusion” appears contradictory to the fact that OPA1 cleavage causes mitochondrial fragmentation by inhibiting fusion in stress conditions. However, if we may offer a reconciliation, the differences in both temporal aspect and cellular energetic state can be taken into account. As for the temporal difference, OPA1 cleavage itself may play a role during the fusion reaction, whereas S-OPA1 is a product of the fusion reaction. Although the mechanism of OPA1-mediated IM fusion is unknown, the IM fusion reaction may require OPA1 cleavage to disassemble OPA1 oligomers. Similar to the Mfn/Fzo1 disassembly that is required for OM fusion, as discussed earlier [47], disassembly of OPA1 oligomers may be necessary for IM fusion as well. Regarding the role of energetic state, although S-OPA1 from the cleavage is fusion incompetent, under normal and energetically active conditions, new OPA1 is continuously synthesized and replenished for subsequent fusion and maintenance of mitochondrial morphology. In contrast, in stressed and energetically deficient conditions, GTP that is necessary for OPA1-mediated fusion may not be available and new protein synthesis is largely inactive. Therefore, OPA1 is cleaved by stress-activated OMA1 without fusion, and fusion-incompetent S-OPA1 accumulates without new OPA1 replenishment, causing mitochondrial fragmentation.

Differential cleavage of OPA1, and thus cell fate, can also be regulated by reciprocal degradation of OMA1 and Yme1L under different ATP levels. It was shown that, in depolarized mitochondria, Yme1L degrades activated OMA1 when ATP level is maintained by glycolysis, whereas activated OMA1 degrades Yme1L when ATP concentration is low [115]. It is possible that, under normal or stress conditions with the ATP levels sufficient for cell survival, Yme1L is active and it removes activated OMA1 to prevent S1 cleavage; however, in pathological conditions with insufficient ATP for cell survival, activated OMA1 would degrade Yme1L, which allows for efficient OMA1-mediated OPA1 cleavage at S1. In addition, Yme1L-independent autocatalytic degradation of activated OMA1 was also reported, in which active OMA1 undergoes self-degradation under stress, presumably to restore OPA1-mediated fusion and mitochondrial integrity upon stress alleviation [111].

Studies with manipulations of OMA1 and Yme1L support the idea that S-OPA1 generated by OMA1-mediated OPA1 cleavage has no significant role in mitochondrial fusion, cristae maintenance, and conferring apoptotic resistance, whereas L-OPA1 can play these roles without S-OPA1. In DKO cells of OMA1 and Yme1L, only L-OPA1 is present due to the complete block of OPA1 cleavage [83]. Mitochondria in these DKO cells were fusion competent and they contained normal cristae. Apoptotic resistance was also observed in the DKO cells. These observations indicate that L-OPA1 alone is sufficient to support fusion, energetics, and cell survival without S-OPA1 [83]. Conversely, KO of prohibitin 2 (PHB2) caused mitochondrial fragmentation due to the selective loss of L-OPA1 and accumulation of S-OPA1 by activated OMA1 [116,117]. PHB2-KO cells also showed disrupted cristae structure and increased susceptibility to apoptotic stimuli. Expressing L-OPA1 in PHB2-KO cells restored tubular mitochondria, normal cristae structure, and apoptotic resistance, whereas S-OPA1 expression failed to do so [116]. Furthermore, OMA1 KO in PHB2-silenced cells stabilized L-OPA1 and increased apoptotic resistance [117]. These observations, together with the observations that were made with OMA1/Yme1L DKO, indicate that L-OPA1 is the functionally active form supporting fusion, cristae maintenance, and apoptotic resistance, whereas S-OPA1 is a nonfunctional proteolytic cleavage product; hence, OPA1 cleavage is detrimental to cell survival.

Several in vivo studies of KO mice for OMA1, Yme1L, and PHB2 also support the notion that excessive OPA1 cleavage is harmful due to the loss of L-OPA1, leading to pathological consequences. Heart-specific KO of Yme1L in mice increased OMA1-mediated OPA1 cleavage and caused dilated cardiomyopathy [118]. As observed in cultured cells, KO of OMA1 in Yme1L-KO hearts stabilized L-OPA1 with no S-OPA1 generation and restored heart function, indicating the positive role of L-OPA1 [118]. KO of PHB2 in forebrain neurons induced a selective loss of L-OPA1 and it caused progressive neurodegeneration, neuroinflammation, and premature death starting at 16 weeks of age [117]. Additional KO of OMA1 in PHB2-KO mice restored L-OPA1, delayed neurodegeneration and neuroinflammation, and extended life span [117]. In another study, it was shown that OMA1-KO prevented OPA1 cleavage in renal ischemia-reperfusion and decreased kidney injury [119]. Additionally, increased levels of mitochondrial ROS were shown to be causal for cristae disruption and cardiac pathology in mouse models of heart failure, and KO of OMA1 decreased heart failure pathology [120].

### 4.5. Potential Caveats Associated with the Existing Paradigm of L- and S-OPA1 Functions

The experimental results that were generated with cells and animals using KOs of OMA1, Yme1L, and PHB2 present evidence that OPA1 cleavage is detrimental due to the loss of L-OPA1 and the accumulation of S-OPA1. These data have also bred the notion that S-OPA1 is a cleavage product bearing no functional significance. However, there have been telltale signs that manipulations of OMA1, Yme1L, and PHB2 may not be entirely appropriate systems for testing the role of L- and S-OPA1. In Yme1L-KO cells, a significant level of L-OPA1 is maintained and yet cristae structure is exceedingly disrupted [83]. On the other hand, cardiac KO of Yme1L had no or minimal effect on cristae structure and respiratory function [118]. In PHB2 KO cells that have only S-OPA1, cristae structure was disrupted, but respiratory function was normal [116]. Furthermore, additional KO of OMA1 in PHB2 KO restored L-OPA1, but failed to restore cristae structure [117]. Therefore, these observations are contradictory to each other and inconsistent with the predictive role of L- and S-OPA1 in cristae maintenance and energetics. One of the issues is that, while OPA1 is cleaved by OMA1 and Yme1L, these proteases, along with m-AAA proteases constitute a part of the mitochondrial quality control system, mediating proteolytic control of the assembly and stability of respiratory complex subunits [121]. Therefore, it is reasonable to assume that KOs of Yme1L and OMA1 would perturb the surveillance mechanism of mitochondrial function, resulting in a pleiotropic effect on mitochondrial structure and function. It should also be noted that OPA1 is not the sole substrate of these proteases [122,123,124,125,126,127,128]. Furthermore, PHB2 has broad functions in other cellular processes in various cellular locations [129,130,131,132]; thus, it is likely that the mitochondrial phenotypes that were observed by KOs of OMA1, Yme1L, or PHB2 include the effects unrelated to OPA1 functions. As such, an experimental system other than the indirect system of manipulating OPA1 cleavage enzymes is desirable in order to define differential roles of L- and S-OPA1.

Another concern is the use of the surrogate S-OPA1 for rescue experiments to test the role of S- and L-OPA1, in which L- OPA1 was found to be the functionally active form, whereas S-OPA1 was nonfunctional [83,116]. This surrogate S-OPA1 is the fusion protein between apoptosis-inducing factor (AIF) and OPA1 called AIF-230. In AIF-230, the N-terminal MTS and TM domain of AIF is fused to the OPA1 part downstream from the S2 cleavage site [73]. Although AIF-230 is correctly targeted to the IM like OPA1 and it contains the “S-OPA1” part, it is predicted to be an IM-anchored protein, because it may not undergo the proteolytic cleavage necessary to generate soluble S-OPA1; therefore, this surrogate TM-containing S-OPA1 may function differently from soluble S-OPA1.

## 5. What’s New for OPA1?

### 5.1. The Role of S-OPA1 in Energetics and Cristae Maintenance

The experimental system that expresses individual OPA1 variants in OPA1 KO cells was initially used to test the differential roles of L- and S-OPA1 in mitochondrial fusion [74]. In two recent reports, this same system was used to differentiate the energetic role of L- and S-OPA1 [96,98]. While these two studies confirmed that S-OPA1 has a minor role, if any, in mitochondrial fusion, new information was obtained for S-OPA1’s role in energetic maintenance [96,98]. Contrary to the prevalent notion that S-OPA1 is the nonfunctional proteolytic product, it was found that S-OPA1 by itself is sufficient to maintain respiratory function independent of L-OPA1 [96,98]. We demonstrated that the expression of a single form of S1-cleaved S-OPA1 in OPA1-KO cells was able to maintain mtDNA, respiration, respiratory complexes, and cristae structure, indistinguishable from that of L-OPA1 [96]. Testing of all eight variants individually also found no distinction among them in energetic activity [98]. These experimental results without manipulating OPA1 cleavage present direct evidence for the function of *bona fide* S-OPA1, demonstrating its energetic competency. Also, this finding that S-OPA1 alone is sufficient for maintaining energetic function, despite lacking fusion activity, supports the previous report that OPA1 function in fusion is mechanistically separate from that of cristae maintenance and energetics [87,90].

The energetic phenotypes of OPA1 deficiency include respiration defects, decreased levels of respiratory complexes and mtDNA, and cristae disruption. When considering OPA1 as being the IM-remodeling protein, it is likely that the energetic defect that is caused by an OPA1 deficiency is due to the lack of adequate cristae structure. Cristae membranes are the assembly platform for respiratory complexes, and therefore, with insufficient cristae, respiratory complexes would become unstable and degraded, which is functionally manifested as a respiration defect. Because mtDNA is attached to the IM, the mtDNA decrease with OPA1 deficiency could also be due to the cristae/IM disruption, although OPA1’s role for mtDNA maintenance was proposed to be dependent on IM fusion function of OPA1 [87]. Regardless, the expression of S-OPA1 restores mtDNA and cristae structure and all related energetic parameters [96,98]. The new finding that S-OPA1 alone, without L-OPA1, is able to support the formation of cristae, as well as the maintenance of cristae tightness, is unexpected but significant, as it prompts the revision of the current paradigm of L- and S-OPA1 functions.

**Figure 4:**
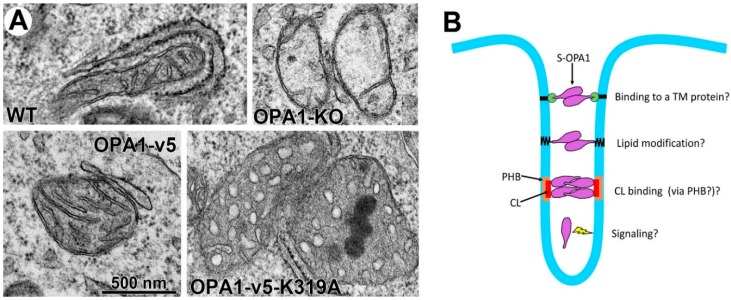
Cristae ballooning by a GTPase-defective OPA1 mutant and potential mechanisms of S-OPA1 for maintaining cristae tightness. (**A**) Transmission EM images of mitochondria. OPA1-KO cells have a few or no cristae. Expression of S-OPA1 (OPA1-v5) in OPA1-KO cells restores cristae structure with tight cristae width. Expression of a GTPase-defective S-OPA1 (OPA1-v5-K319A) in OPA1-KO cells restores cristae density, but not cristae tightness. (**B**) S-OPA1 may maintain cristae tightness by (1) binding to a TM-anchored protein, (2) IM-anchoring itself by lipid modification, or (3) binding to local CL that may be enriched by prohibitin (PHB). A signaling function of S-OPA1 is also possible.

OPA1-KO cells show no or very few cristae, and we found that expression of either L- or S-OPA1 increased cristae number, indicating that OPA1 is necessary for cristae formation [96], though it is currently unknown how OPA1 mediates this process. Most strikingly, however, when we tested the GTPase-defective OPA1 mutants, all cristae were swollen and round, indicating a loss of cristae tightness (Figure 4A, OPA1-v5-K319A), regardless of whether the mutant was L- or S-OPA1 form [96]. An interesting fact is that the mutant OPA1 cells show the presence of numerous cristae, despite their ballooned shape. These observations demonstrate that OPA1 GTPase activity is dispensable for cristae formation, but are necessary for maintaining cristae tightness. The restoration of cristae number by GTPase-defective OPA1 suggests that the presence of OPA1 molecules, regardless of their GTPase activities and L or S form, is sufficient for IM expansion/reorganization that are necessary for cristae formation. Another interesting finding is that the GTPase-defective L- or S-OPA1 mutant is unable to support complex V maturation, while the wild-type versions are [96]. In OPA1-KO cells and cells expressing GTPase-defective L- or S-OPA1, a significant amount of F_1_ was detected without assembly into the mature F_1_F_o_-ATP synthase complex [96]. Dimerization and oligomerization of complex V are known to be involved in cristae shaping [6,12,13,133]. Possibly, the lack of complex V assembly in OPA1 mutant cells could cause the loss of cristae tightness.

The current model states that L-OPA1 molecules anchored in the opposing cristae membranes trans-interact to keep cristae tight [90,107,108], which cannot be supported by TM-free soluble S-OPA1. If this model is still valid and applicable for the newly found S-OPA1-mediated cristae tightness, membrane anchoring of S-OPA1 would be required. It is possible that S-OPA1 interacts with a membrane-anchored protein to assume an L-OPA1-like conformation (Figure 4B). Alternatively, S-OPA1 may undergo a lipid modification to anchor itself in the membrane (Figure 4B). Local lipid composition is likely to play a role in cristae shaping. As S-OPA1 binds to CL [61,108], increased local CL concentration may allow for S-OPA1 membrane binding and oligomerization, which might support cristae tightness. Prohibitins (PHB1 and PHB2) are known to form ring complexes in the IM to define local membrane domains [134]. S-OPA1 may require PHB1/2 for membrane binding and cristae maintenance (Figure 4B). Although OPA1 is associated with the cristae membrane and it can bind and remodel membranes in vitro, there is no direct structural evidence that supports the current model of cristae width maintenance by forming “bridge” or “staple”-like structure. It was proposed that conventional dynamin can function as a signaling GTPase [135,136]. The possibility for OPA1 as a signaling molecule instead of a structural protein cannot be ruled out. This potential signaling function may not require IM-anchoring of the OPA1 protein (Figure 4B).

### 5.2. New Understanding of OPA1 Cleavage

S-OPA1, the cleavage product, has now been shown to be functionally replaceable for L-OPA1 in its energetic maintenance, providing a new meaning for OPA1 cleavage. In terms of IM fusion, OPA1 cleavage may have different functions and consequences, depending on cellular energetic states, as discussed earlier (Section 4.4). Under energetic sufficiency, OPA1 cleavage was shown to be a driving event for IM fusion [110]. Possibly, OPA1 cleavage may have a similar stimulatory effect for energetic maintenance under normal non-stress conditions. Although the fusion function of OPA1 is mechanistically independent of its crista maintenance role, the two processes may not be entirely separate functionally. Increasing OPA1 level augments both fusion and energetic activity [97,137,138], suggesting that there may be a functional link between mitochondrial fusion and crista maintenance. One can envision that, following the fusion of two structurally and functionally distinct mitochondria, IM reorganization is necessary for functional optimization of the newly fused mitochondria. The membrane-anchored L-OPA1 is likely restricted only to two-dimensional movement in the IM. On the other hand, soluble S-OPA1 would be trans diffusible in the IMS and thus able to expedite crista reorganization of newly fused mitochondria. It is possible that mitochondrial fusion and IM remodeling are coupled events, in which IM fusion generates diffusible S-OPA1 by OPA1 cleavage that facilitates post-fusion IM remodeling.

A similar link between mitochondrial fusion and energetic maintenance can be considered in stress conditions. OMA1 is activated under cellular stress and cleaves OPA1, which blocks fusion and thus causes mitochondrial fragmentation. Mitochondrial fragmentation is thought to be detrimental, leading to apoptosis by facilitating cytochrome *c* release [139,140,141]. Then, the question is why cells activate OMA1 under cellular stress for OPA1 cleavage. Is it simply to cause mitochondrial fragmentation and kill themselves? It may be, if OPA1 cleavage inactivates OPA1 function. However, we now know that OMA1-mediated OPA1 cleavage generates S-OPA1 that is capable of supporting energetic function, albeit lacking in fusion activity [96,98]. Furthermore, mitochondrial fragmentation can be protective, because it can segregate dysfunctional mitochondria by fusion inhibition for autophagic removal [112,113] and can prevent the spreading of harmful factors such as local Ca^2+^ overload [142]. Accordingly, controlling mitochondrial shape and energetic activity under stress conditions can also be linked by OPA1 cleavage. Under stress conditions, S-OPA1 generation by OPA1 cleavage induces mitochondrial fragmentation by fusion inhibition, which would be beneficial, as mentioned above. At the same time, S-OPA1 would support energetics in functional mitochondria under cellular stress. Autophagic recycling of dysfunctional mitochondria may as well support the substrate supply for mitochondrial energetics. In this context, stress-induced OPA1 cleavage may be a previously unrecognized cellular response to extend cell survival in cells facing adverse conditions.

## 6. Concluding Remarks

Mitochondrial dynamins mediate fission and fusion of mitochondrial membranes to change mitochondrial shape. Studies for understanding the mechanisms of fission and fusion have mainly been focused on the role of protein factors. However, dynamins are membrane-remodeling proteins, and thus lipid molecules are important factors in fission and fusion processes. In this review, in addition to a brief overview of the role of the fission and fusion proteins, we have had an opportunity to discuss the small but significant progress that was recently made in the role of lipids in mitochondrial dynamics. Through the studies of mitochondrial shape change in physiology and pathology, it has become evident that mitochondrial morphology and energetic activity are closely linked. OPA1 is a unique protein that regulates both mitochondrial shape and energetics by remodeling the IM. The proteolytic cleavage of OPA1 has drawn particular attention due to its association with structural and functional changes of mitochondria in cellular stress and pathology. The main paradigm has been that the proteolytic cleavage of L-OPA1 generates and accumulates S-OPA1, which is incompetent for fusion and energetic maintenance, leading to mitochondrial and cell injury. However, this paradigm has recently been challenged by a new finding that S-OPA1 has the capacity of maintaining energetic activity, which prompts a fundamental shift in the idea about OPA1 cleavage. As discussed, OPA1 cleavage may play a role in coupling mitochondrial shape change with the regulation of energetic activity and may represent a novel cellular survival mechanism in cells under stress. While intriguing, this new idea of OPA1 cleavage will need further evaluation and experimental corroboration.
